# The Effects of Controlled Release of Neurotrophin-3 from PCLA Scaffolds on the Survival and Neuronal Differentiation of Transplanted Neural Stem Cells in a Rat Spinal Cord Injury Model

**DOI:** 10.1371/journal.pone.0107517

**Published:** 2014-09-12

**Authors:** Shuo Tang, Xiang Liao, Bo Shi, Yanzhen Qu, Zeyu Huang, Qiang Lin, Xiaodong Guo, Fuxing Pei

**Affiliations:** 1 Department of Orthopaedics, West China Hospital, Sichuan University, Chengdu, China; 2 Department of Pain Medicine, Shenzhen Nanshan Hospital, Shenzhen, China; 3 Department of Orthopaedics, Mianyang Center Hospital, Mianyang, China; 4 Department of Orthopaedics, Union Hospital, Tongji Medical College, Huazhong University of Science and Technology, Wuhan, China; 5 Department of Orthopaedics, Guangdong hospital of traditional Chinese medicine, Guangzhou, China; Temple University School of Medicine, United States of America

## Abstract

Neural stem cells (NSCs) have emerged as a potential source for cell replacement therapy following spinal cord injury (SCI). However, poor survival and low neuronal differentiation remain major obstacles to the use of NSCs. Biomaterials with neurotrophic factors are promising strategies for promoting the proliferation and differentiation of NSCs. Silk fibroin (SF) matrices were demonstrated to successfully deliver growth factors and preserve their potency. In this study, by incorporating NT-3 into a SF coating, we successfully developed NT-3-immobilized scaffolds (membranes and conduits). Sustained release of bioactive NT-3 from the conduits for up to 8 weeks was achieved. Cell viability was confirmed using live/dead staining after 14 days in culture. The efficacy of the immobilized NT-3 was confirmed by assessing NSC neuronal differentiation in vitro. NSC neuronal differentiation was 55.2±4.1% on the NT-3-immobilized membranes, which was significantly higher than that on the NT-3 free membrane. Furthermore, 8 weeks after the NSCs were seeded into conduits and implanted in rats with a transected SCI, the conduit+NT-3+NSCs group achieved higher NSC survival (75.8±15.1%) and neuronal differentiation (21.5±5.2%) compared with the conduit+NSCs group. The animals that received the conduit+NT-3+NSCs treatment also showed improved functional outcomes, as well as increased axonal regeneration. These results indicate the feasibility of fabricating NT-3-immobilized scaffolds using the adsorption of NT-3/SF coating method, as well as the potential of these scaffolds to induce SCI repair by promoting survival and neuronal differentiation of transplanted NSCs.

## Introduction

Trauma-induced spinal cord injuries (SCI) resulting in significant motor impairment or paralysis remain a critical public health concern with approximately 100,000 new cases each year [1]. SCI interrupts connections between the brain and the spinal cord, which transmit motor control and somatic sensory signals. Victims of SCI often suffer from severe neurological disabilities. However, effective treatment is currently limited because of the complexity of the pathophysiology of the injured spinal cord. SCI triggers a series of events, including systemic and local inflammatory responses, subsequent death of neuronal and glial cells, and formation of cavities and glial scars in the injury site [2].

Cell transplantation therapy provides a potential source of cells to repopulate the damaged spinal cord and aid in functional recovery by replacing damaged circuits, increasing plasticity, and promoting cell survival and regeneration of host axons [3]. Neural stem cells (NSCs) have the potential to proliferate, migrate, and differentiate into the three major cell types of the central nerve system, oligodendrocytes, astrocytes, and neurons, to replace lost cells or rescue dysfunctional cells [4], [5]. However, poor cell survival and uncontrolled differentiation of transplanted NSCs are the current limitations of this approach [6]. Transplanted NSCs have a much greater tendency to astrocytically differentiate, and they rarely undergo neuronal differentiation. Neurons that have differentiated from transplanted NSCs can extend axons into the host spinal cord in both rostral and caudal directions over remarkably long distances, and connections with the host axons are then formed. The host axons can also regenerate into the sites of transplanted NSCs and promote host-to-graft connectivity [7]. The differentiation into oligodendrocytes can promote remyelination of regenerated axons [8]. However, the differentiation of transplanted NSCs into astrocytes may be problematic because reactive gliosis is believed to be an inhibitor of regeneration [9]. Therefore, increasing the rate of survival and differentiation of NSCs into neuronal cells is important.

Neurotrophins (NTs), such as nerve growth factor (NGF), brain-derived neurophic factor, and neurotrophin-3 (NT-3), are important in neuronal survival and differentiation [10], [11]. Among these factors, NT-3 facilitates the differentiation of NSCs into neurons and supports the survival and maturation of neurons [12]. Combining NSC transplantation with NT-3 is considered a potentially useful approach for the treatment of SCI [13]. NSC transplantation and soluble NT therapies are limited by the poor survival of injected cells and the short half-life of injected NTs. The external pump/catheter system is used as a controlled release system for delivering drugs into the intrathecal space through a catheter. However, this method is prone to infection and has not been approved for long-term delivery in SCI patients in the USA [14]. To address these issues, tissue-engineered scaffolds have been developed for the treatment of SCI. Biodegradable scaffolds and NSC transplantation each have unique advantages as therapeutic strategies for SCI. Biodegradable scaffolds have many advantages as a delivery system, not only for sustained NT-3 delivery but also for the delivery of NSCs to the injured spinal cord [15]. Furthermore, scaffolds can also bridge the lesion with a 3D permissive environment, which can fill the tissue gap and concomitantly support axonal regeneration [16].

Thus, the incorporation of NT-3 within a biodegradable scaffold offers an opportunity to promote the survival and differentiation of NSCs. Most of the current approaches in incorporating growth factors in biomaterials include physically entrapping soluble growth factors into the scaffolds or covalently immobilizing growth factors onto synthetic polymer scaffolds [17]. By immobilizing growth factors within scaffolds, the growth factors are protected against cellular inactivation and digestion [18]. As a result, the immobilized growth factors can overcome the diffusional limitations of soluble factors, thereby allowing sustained activity. However, most of the covalent immobilization methods require the use of organic solvents or cross-linking agents, which may limit the biological activity of the growth factors. Furthermore, by using these methods, the immobilization of growth factors is not a simple task [19].

Silk fibroin (SF), a natural protein, has been recently found to be biocompatible, slowly biodegradable, and endowed with excellent mechanical properties and processability [20]. SF matrices can successfully deliver growth factors while preserving their potency [21]. Interestingly, SF in aqueous solution can precipitate due to β-sheet formation. SF adsorbs onto various substrates spontaneously and forms a layer, which eventually forms a robust and stable coating through mild all-aqueous processes [22]. Growth factors can be incorporated into the SF matrix during these processes. With the degradation of SF, growth factors can be released. In our previous study, Poly(ε-caprolactone)-block-poly(l-lactic acid-co-ε-caprolactone) (PCLA) scaffolds with precise hierarchical pore architectures were fabricated using injection molding combined with thermally induced phase separation [23]. In the present study, the PCLA scaffolds (membranes and conduits) were fabricated first, and then NT-3 was immobilized within the scaffolds by coating the scaffold with a solution of SF and NT-3. NSCs were cultured in vitro, and their differentiation into neural cells was measured after seeding on the NT-3-immobilized membranes. A rat spinal cord transection model was utilized to evaluate the efficacy of the NT-3 immobilized conduit with the adhered NSCs in vivo.

## Materials and Methods

### Ethics Statement

All experimental protocols were approved by the Ethics Committee in Sichuan University (protocol # 2013-C-206). The animals were maintained on a standard laboratory diet in plastic cages at ambient temperature, and allowed free mobilization in compliance with the requirements of the International Council on Laboratory Animal Science.

### Scaffold fabrication and NT-3 immobilization

PCLA: [PCL27-b-P(LLA405- co-CL14) (PCLA); *Mn*  =  8.8×10^4^, *PDI* = 1.22, *T* = 171.2°C, and *ΔH* = 39.3 J/g. PCLA was first dissolved in dioxane with a concentration of 60 mg/ml at 50°C, and the mold was chilled to the preset temperature of −40°C. The hot PCLA/dioxane solution was quickly injected into the cold mold using a syringe, as previously described [24]. The temperature of the system was maintained at −40°C for 2 h to induce the solid-liquid phase separation of the polymer solution. Then, the temperature of the cold trap was adjusted to −20°C, and the solvents were sublimated through a two-day lyophilizing process. Finally, the mold was removed, and the 2D scaffolds (membranes) and the 3D scaffolds (conduits) for in vitro and in vivo studies, respectively, were obtained. The prepared conduit had an inner diameter of 3.0 mm, a wall thickness of 0.5 mm and a length of 2.0 mm.

The SF aqueous solution was prepared as previously described [22]. Briefly, cocoons from *Bombyx mori* were boiled in ultra-purified water (UPW) containing 0.02 M Na_2_CO_3_, thoroughly rinsed with distilled water to extract the glue-like sericin proteins and wax, and then dissolved in 9 M LiBr at 55°C to obtain a 3% (w/v) aqueous SF solution. NT-3 (PeproTech, Rocky Hill, NJ, USA) was embedded by adding 20 µg of NT-3 to 1 ml of SF solution. The NT-3/SF solution was subsequently sterilized using a 0.22 µm filter (Millipore, Tullagreen, Ireland). The scaffolds were immersed in the NT-3/SF solution for 2 h at room temperature. Then, the scaffolds were freeze-dried overnight and subsequently treated with 90% (v/v) methanol in UPW for 15 min to induce formation of SF crystalline β-sheet structure. The scaffolds were stored in a desiccator and rinsed with sterile PBS three times before use.

### Determination of kinetics of NT-3 release from NT-3 immobilized PCLA scaffold

Five conduits were separately immersed in 500 µl PBS (10 mM, pH 7.4) and kept in a gently shaking incubator (20 rpm) at 37°C for various time periods up to 8 weeks. The release of NT-3 was examined on day 1, with further measurements after 2, 3, 4, 5, 6, and 7 days. After that, continuous measurements were performed for 8 weeks at 1 week intervals. At each time interval, the supernatant was removed completely and replaced with fresh buffer. The amounts of NT-3 in the collected supernatants were measured using enzyme-linked immunosorbent assay (ELISA; Becton Dickinson, Franklin Lakes, NJ, USA) in accordance with the manufacturer's instructions. The absorbance was measured at 450 nm using a plate reader (Model 680; Bio-RAD, Hercules, CA, USA). The NT-3 concentration was determined by comparing the reading to the standard curve. The amounts of NT-3 in each conduit were calculated based on the cumulative amounts of bioactive NT-3 released in PBS and normalized by the mass of the conduit.

### Isolation and culture of rat brain-derived NSCs

Primary NSCs were isolated from E14 Sprague-Dawley (SD) rats, expressing green fluorescent protein (GFP) for the in vivo study and non-GFP for the in vitro study. The transgenic SD rats expressing GFP were purchased from Cyagen Biotechnology Company Ltd. (Guangzhou, China). Whole hippocampi were dissected and dissociated in Hanks' balanced salt solution (HBSS). After centrifugation at 1000 rpm for 5 min, the supernatant containing cell debris was removed. The pelleted cells were resuspended in growth medium, which contained Neurobasal media (Gibco-Invitrogen, Carlsbad, CA, USA), B27 neural supplement (Gibco-Invitrogen), 2 mM L-glutamine (Sigma-Aldrich, St Louis, MO, USA), 1% penicillin-streptomycin (Sigma-Aldrich), 20 ng/ml epidermal growth factor (Gibco-Invitrogen), and 20 ng/ml bFGF (Gibco-Invitrogen). The cells were then plated onto 75 ml culture flasks, with fresh medium every 3 days. The cultured cells typically grew as suspending neurospheres and were passaged once a week. The expression of nestin, a marker of NSCs, was assessed by immunocytochemistry.

### PCLA membrane studies

PCLA membrane were placed in 24-well plates and sterilized with 70% ethanol for 5 min, followed by washing three times with PBS solution prior to use. NSCs were spun down at 1500 rpm for 5 min, and the resultant pellet was re-suspended and dissociated in fresh growth media. The cell solution was then added so that each well contained 10 000 cells, and the final volume of media in the wells was 500 µl. After 24 h, the media was removed and replaced with differentiation media – Neurobasal media containing B27 supplement, L-glutamine, penicillin-streptomycin, and 1% fetal bovine serum (FBS). Half-volume media changes were performed every 48 h thereafter.

The viability of cells on the membranes was determined using a live/dead assay (Molecular Probes, Eugene, OR), in accordance with the manufacturer's protocol. Briefly, after 14 days of culture, the PCLA membranes were rinsed with 0.1 M PBS (pH 7.4) three times, followed by staining in 2 ml 0.1 M PBS containing 2 mM of calcein-AM and 4 mM ethidium homodimer (EthD-III) for 30 min at 37°C. The cells were washed again with PBS and then imaged using an Olympus fluorescent microscope. Live cells stained with calcein-AM showed a green color, and dead cells stained with EthD-III showed a red color. Cell viability was calculated by counting the percentage of calcein-AM-positive cells over the total number of cells from five randomly chosen fields of view per sample.

Differentiation was determined using immunocytochemistry staining following the standard protocol. The following primary antibodies were used for immunohistochemical analysis: rabbit anti-2',3'-cyclic nucleotide 3'-phosphodiesterase (CNPase, 1∶100, Cell Signaling Technology, Beverly, MA), mouse anti-glial fibrillary acidic protein (GFAP; 1∶500, Sigma, St. Louis, MO) for astrocytes, and rabbit anti-microtubule-associated protein 2 (MAP2; 1∶500, Sigma, St. Louis, MO) for neurons. Briefly, the samples were fixed in 4% paraformaldehyde for 20 min at 4°C and then permeabilized with 0.3% Triton X-100 for 5 min. Non-specific binding was blocked with 10% goat serum and 1% bovine serum albumin (BSA) for 1 h at room temperature. The samples were subsequently incubated with the primary antibodies overnight at 4°C. After washing with PBS, the samples were incubated at room temperature for 2 h with secondary fluorescent antibodies: Alexa Fluor 488-conjugated goat anti-rabbit IgG or Alexa Fluor 594-conjugated goat anti-mouse IgG (1∶500, Invitrogen, Carlsbad, CA, USA). The nuclei were stained with 4',6-diamidino-2-phenylindole (DAPI; Sigma-Aldrich, St Louis, MO, USA). The samples were viewed under an Olympus fluorescent microscope. Cell differentiation was calculated by counting the percentage of MAP2-, GFAP- and CNPase-positive cells from five randomly chosen fields of view per sample using ImageJ software (National Institutes of Health, Bethesda, MD).

### Seeding of NSCs into PCLA conduits

Sterile conduits were incubated in complete media overnight, prior to cell seeding. Before seeding the NSCs into the conduits, the NSCs were digested into single cell suspension. The media inside the tubes were then replaced with 80 µl of cell suspension, which contained GFP-positive cells (passage 4) at a cell density of 4×10^6^ cells per 80 µl. For the *in vivo* study, the conduits containing the neurosphere suspension were rotated manually every 15 min for 1 hour to achieve uniform cell seeding. These conduits were then transferred to new wells, which contained growth media, and incubated for 24 h prior to implantation into the transected spinal cords. Scanning electron microscopy (SEM, JSM-5800LV) (JEOL, Tokyo, Japan) was used to observe the cell morphology after 24 h of culture. The cell density inside the channels was not uniform and covered mainly the lower half of the channels due to gravity.

### Animal surgery and post-operative care

All of the animal experiments were approved and performed according to the regulations of the Animal Ethical Committee of our university. Adult male rats (220–250 g, purchased from the Experimental Animals Center of Sichun University) were divided randomly into three groups. 1, conduit+NT-3+NSCs group (*n* = 8): the transected spinal cord was bridged by an NT-3 immobilized conduit with NSCs; 2, conduit+NSCs group (*n* = 7): the transected spinal cord was bridged by a conduit with NSCs; 3, conduit+NT-3 group (*n* = 7): the transected spinal cord was bridged by an NT-3 immobilized conduit without NSCs. Each rat was anesthetized with an intraperitoneal injection of sodium pentobarbital (50 mg/kg) before the operation. We performed all of the surgical procedures under a microscope and under sterile conditions. The backs of rats were shaved and aseptically prepared. After checking of the lowest lumbar level, a laminectomy was performed to expose the T8-10 spinal segments. The posterior aspect of the spinal cord was exposed and the dura was cut vertically using microforceps and microscissors. An angled microscissor was used to transect the spinal cord competely and the stumps were retracted, creating a 2.0-mm gap in the spinal cord of T9. To prevent unexpected reflex movements and bleeding, we applied two drops of 1% lidocaine with epinephrine onto the spinal cord. The conduit was grafted into the lesion site in a manner such that the cut ends of both spinal stumps were apposed to the NSCs-containing conduit. The exposed spinal cord with the implanted conduit was covered with muscles and fascia. The skin was repositioned and closed with sutures.

After surgery, the rats were injected with 5.0 ml saline subcutaneously and allowed to recuperate. Buprenorphine (0.03 mg/kg) was administered subcutaneously for 3 days to minimize pain. Enroflaxacin (2.5 mg/kg) was administered subcutaneously for 7 days for prophylactic treatment against postoperative infections. Bladders were manually expressed thrice daily for the duration of the experiment. Functional recovery was assessed weekly during the 8 week survival period using the Basso, Beattie, Bresnahan (BBB) open field locomotor scale [25]. BBB scoring was conducted by two observers blinded to the experimental groups. All of the animals were evaluated weekly for 8 weeks.

### Tissue preparation and cell counts of GFP-positive cells

The animals were sacrificed via transcardial perfusion with 4% paraformaldehyde in 0.1 M PBS at 8 weeks after implantation. The spinal cords were carefully removed, postfixed in 4% paraformaldehyde for 5 h, and then transferred to 0.1 M PBS containing 30% sucrose at 4°C overnight. Afterwards, they were frozen and embedded in an optimal cutting temperature (OCT) compound (Sakura, Tokyo, Japan), and then cut into 15 µm sections on a freezing microtome for cell count and histological analysis.

To quantify cell survival, every tenth equidistant frozen section of the spinal cord was selected from each animal and counterstained with the nuclear dye DAPI. The samples were examined using a Zeiss LSM 710 laser scanning confocal microscope (LSCM; Zeiss, Oberkochen, Germany). A minimum of five random fields containing the GFP-positive transplanted NSCs were photographed over multiple tissue sections of each animal. Cell survival was calculated by manually counting GFP-positive cells associated with DAPI stained nuclei across the sample.

### Histological analysis

In addition to the antibodies previously described, mouse anti-neurofilament 200 (NF200; 1∶500, Sigma, St. Louis, MO) for axons was also used. The secondary antibodies were Alexa Fluor 594-conjugated goat anti-mouse or goat anti-rabbit IgG antibodies (1∶500, Invitrogen, Carlsbad, CA, USA). The sections were washed with PBS and permeabilized with 0.3% Triton X-100 for 5 min. Non-specific binding was blocked using 10% goat serum and 1% BSA for 1 h at room temperature. After washing with PBS, the sections were incubated in primary antibody overnight at 4°C. Afterwards, sections were washed with 0.1 M PBS, and corresponding secondary antibodies was added for 1 h at 25°C. After another series of washes, the nuclei were stained with DAPI. Finally, the immunostained samples were mounted onto glass slides with mounting medium and viewed under the LSCM. In all the immunohistochemistry procedures, appropriate negative controls were used without the primary antibodies.

For histological analysis, every tenth equidistant frozen section of the spinal cord was selected from each animal. A minimum of five random fields containing the GFP-positive transplanted NSCs were photographed per sample. Phenotypic analysis of transplanted NSCs was calculated based on identification of GFP-positive cells associated with a DAPI stained nucleus and the immunohistological marker of interest. The number of NF200-positive axons in the conduits was also counted.

### Statistical analysis

Analysis of variance (ANOVA) was used to test for the statistical significance, and the results were accepted when two-tailed *P<*0.05. Tukey's post-test was performed to compare individual pairs of means if ANOVA *P*<0.05. The results are presented as mean ± standard deviation.

## Results

### Isolation and culture of NSCs

**Figure 1:**
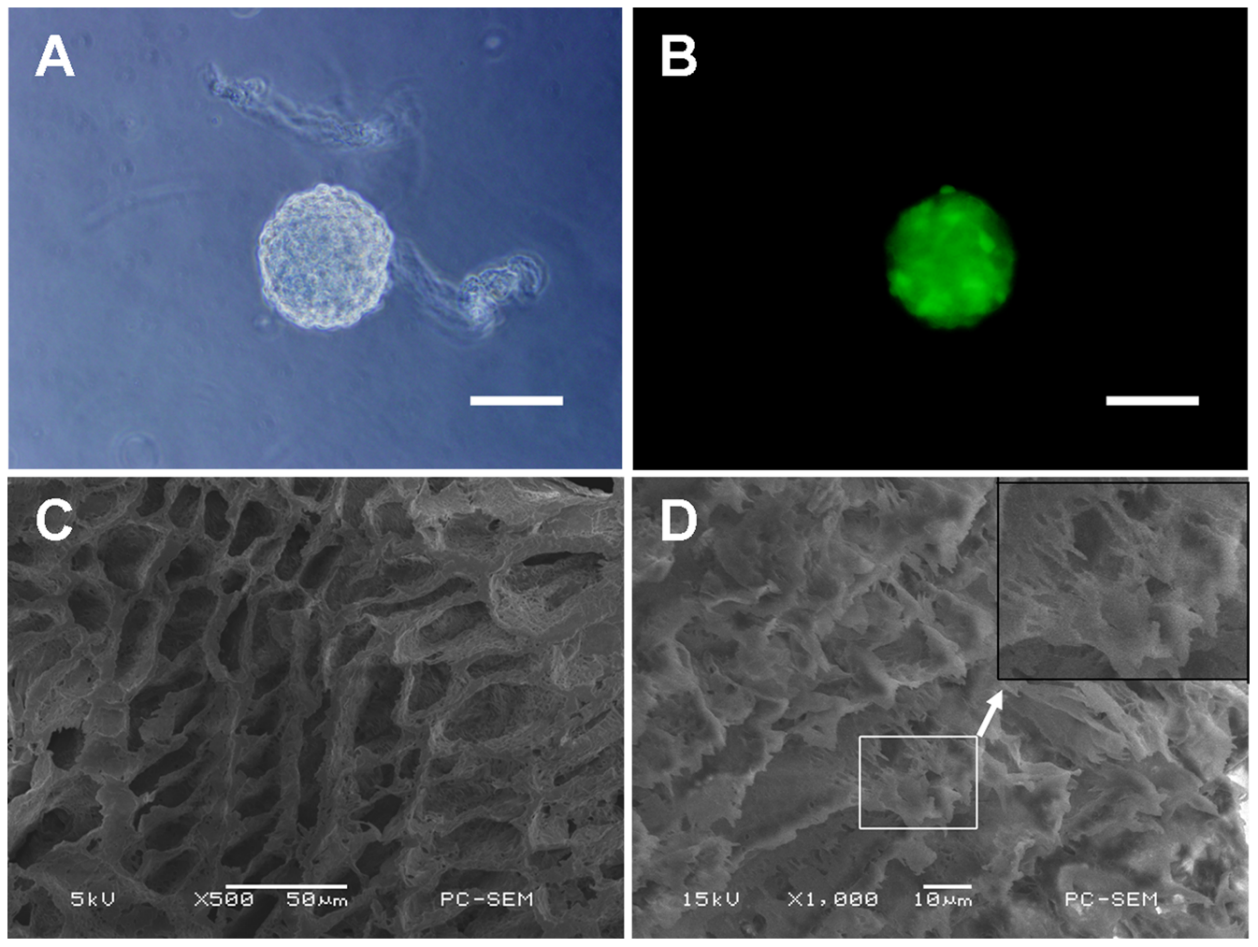
Neurosphere and PCLA scaffold. (A) A neurosphere visualized under a light microscope. (B) GFP-positive neurosphere visualized under a fluorescent microscope. (C) SEM images of the inner wall of the PCLA conduit without NSCs. (D) NSCs adhered to the inner wall of the PCLA conduit. The black box show representative cells from the white box at higher magnification to enhance visualization of cellular morphology under SEM. Scale bars =  100 µm in A–B, 50 µm in C, and 10 µm in D.

NSCs were isolated from the whole hippocampi of E14 SD rats. The NSCs aggregated as free-floating neurospheres (Fig. 1A), as observed under a phase contrast microscope after 5 days of culture. The formation of neurospheres is considered the gold standard for the identification of NSCs. Then, the NSCs were assessed using nestin, a marker for neural precursors. The immunocytochemistry results indicated that the neurospheres showed positive immunoreactivity to nestin, a known protein marker of NSCs (Fig. 1B). These data indicated that NSCs maintained their undifferentiated status in the defined medium. The NSCs grafted into the NT-3-immobilized PCLA conduits were shown in Fig. 1C and D.

### Determination of kinetics of NT-3 release from NT-3 immobilized scaffolds

**Figure 2:**
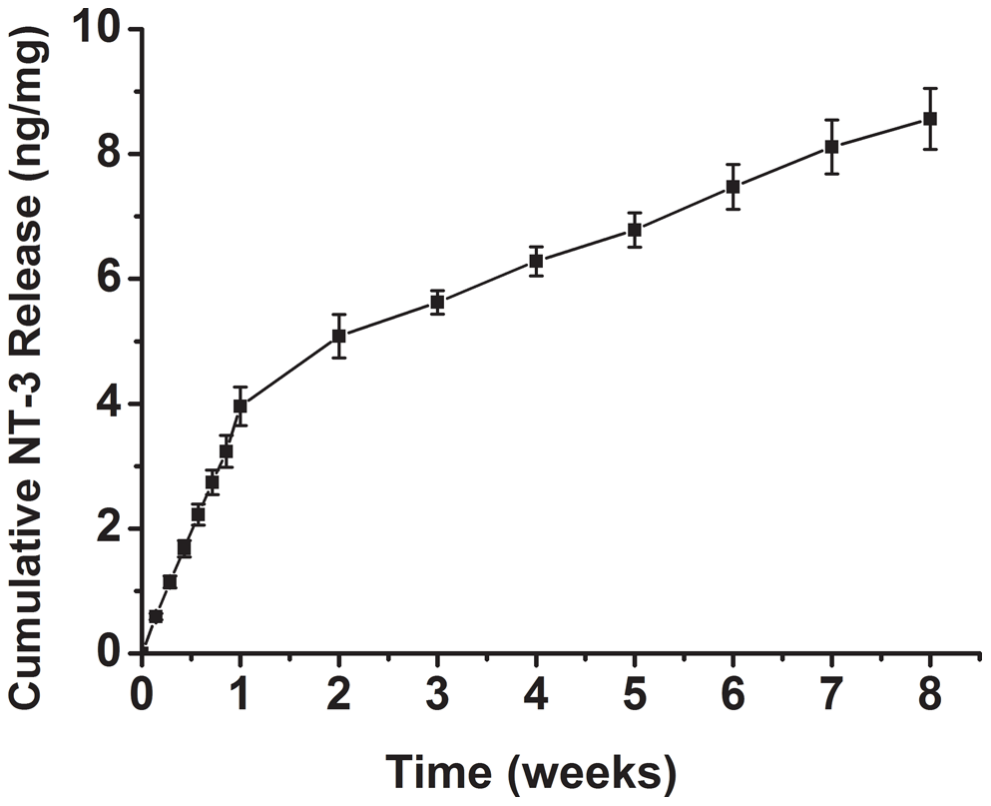
Cumulative release profile of bioactive NT-3 from NT-3-immobilized PCLA-SF conduits in vitro. n = 3.

NT-3 was released from NT-3-immobilized conduits in vitro for at least 8 weeks (Fig. 2). The release profile was composed of two parts, an initial burst in the first week followed by a sustained release. After an initial burst release in the first week, the NT-3-immobilized conduits released NT-3 at a stable and constant rate in the following seven weeks. The cumulative amount of released NT-3 was 8.56 ng/mg.

### Viability and differentiation of NSCs on NT-3-immobilized PCLA membranes

**Figure 3:**
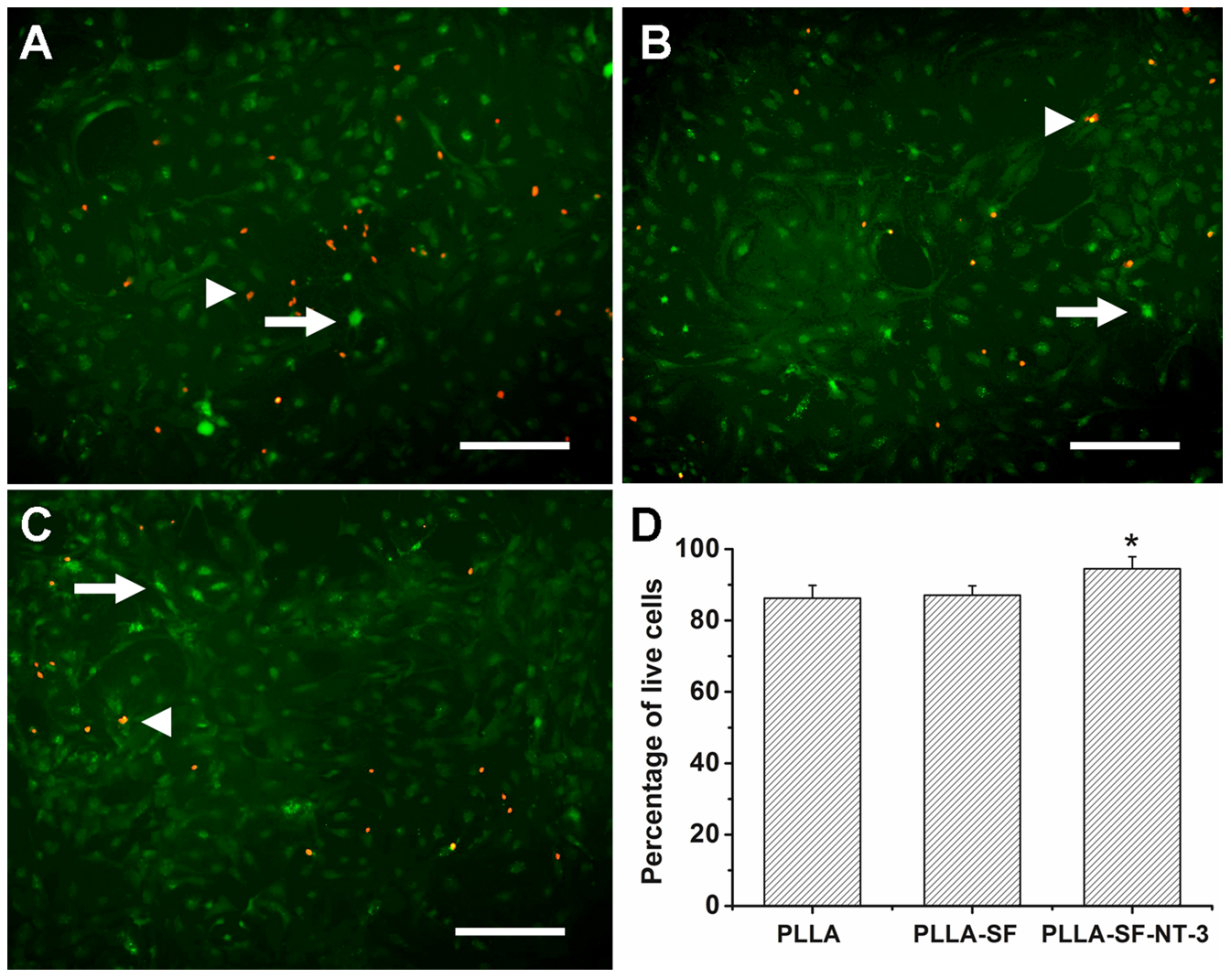
Fluorescent staining for cell viability of NSCs grown on PCLA (A), PCLA-SF (B), and PCLA-SF-NT-3 (C) membranes for 14 days. Living cells (white arrows) were in green and dead cells (white arrowheads) were in red. (D) The percentage of live cells. Scale bars = 100 µm; *P<0.05; n = 4.

To determine the viability of the NSCs on the membranes, the seeded cells were cultured for 14 days, and then stained with calcein-AM and EthD-III. The cell viability on PCLA-SF-NT-3, PCLA-SF, and PCLA membranes were determined to be 94.52±3.37%, 87.12±2.63%, and 86.86±3.61%, respectively (Fig. 3A–D). The percentage of live cells was highest on PCLA-SF-NT-3 membranes. No significant difference in viability was found between the PCLA-SF and PCLA membranes.

**Figure 4:**
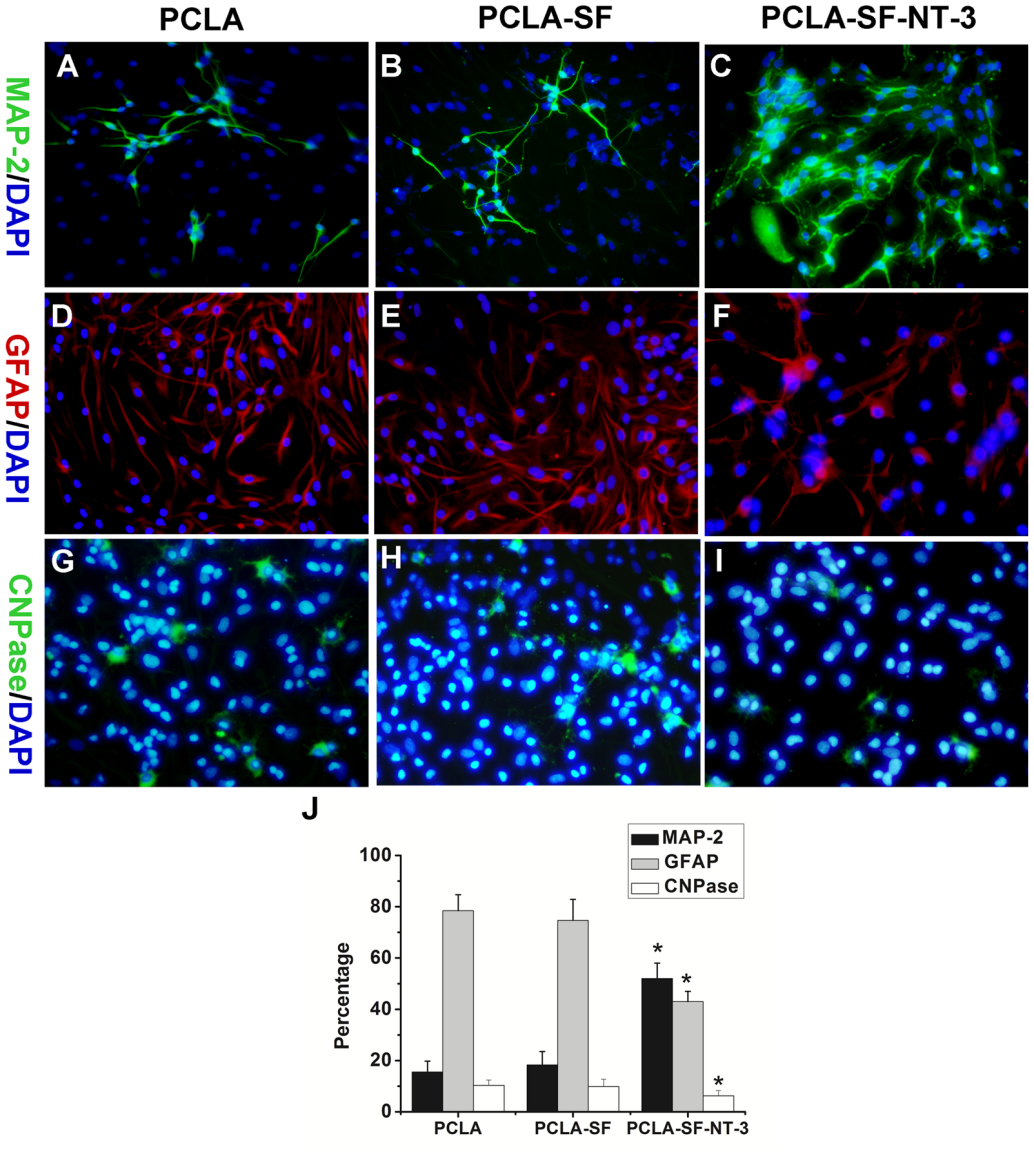
Differentiation of NSCs in vitro. (A–I) Cells cultured on PCLA (A, D, G), PCLA-SF (B, E, H), and PCLA-SF-NT-3 (C, F, I) membrane were immunostained with MAP-2 for neurons (A–C), GFPA for astrocytes (D–F), CNPase for oligodendrocytes (G–I). The nuclei were stained with DAPI. (J) Quantitative analysis of the NSC differentiation in vitro. Scale bars = 25 µm, *P<0.05; n = 4.

To evaluate the differentiation of seeded NSCs on the NT-3-immobilized PCLA membranes, we stained the cells with MAP-2, GFAP, and CNPase (Fig. 4A–I). At day 14, the percentages of MAP-2-positive cells on PCLA, PCLA-SF, and PCLA-SF-NT-3 membranes were determined to be 18.6±2.2%, 25.3±3.2%, 55.2±4.1%, respectively. The percentages of GFAP-positive cells and CNPase-positive cells were the lowest in the PCLA-SF-NT-3 group, compared with the PCLA and PCLA-SF groups (Fig. 4J).

### Long-term survival and differentiation of NSCs after SCI

**Figure 5:**
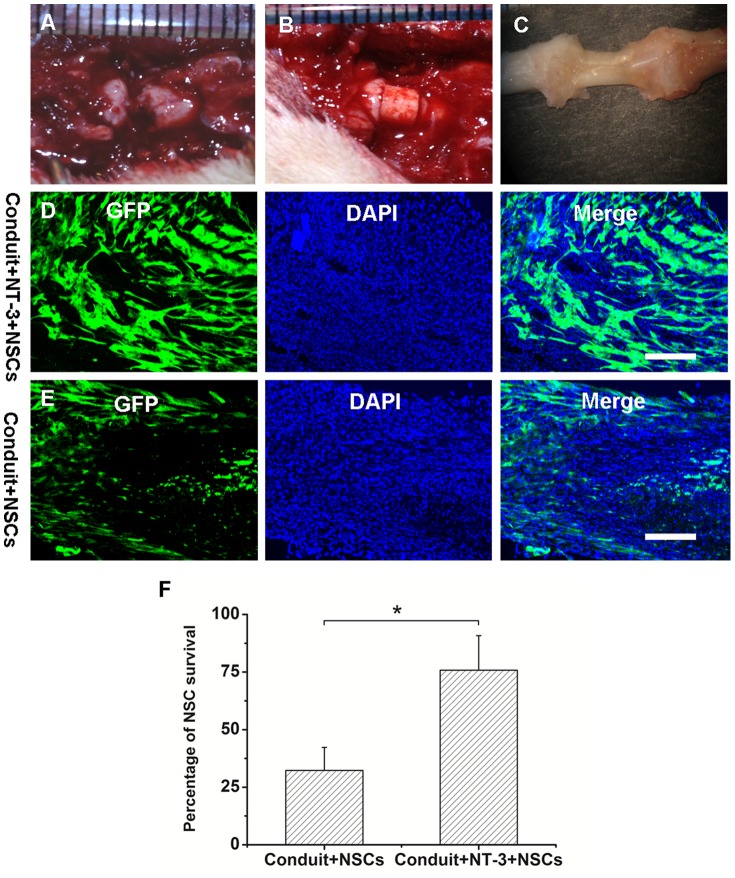
Conduit implantation after spinal cord transection facilitates tissue bridging and NSC survival. (A–B) Surgical procedure for implantation of conduits into completely transected rat spinal cords. (C) Gross appearance of spinal cords 8 weeks after implantation of the conduits. (D–E) Confocal fluorescent images of NSC survival in the conduit+NT-3+NSCs (D) and conduit+NSCs (E) groups 8 weeks after implantation. (F) Percentage of NSC survival for each group. Scale bar  =  100 µm; *P<0.05; n = 7.

The surgical procedures are shown in Fig. 5A and B. During the experiment, no rats exhibited wound complications. At explantation, no inflammatory signs or adverse tissue reactions were observed. General observation of both ends of the lesion site revealed reparative tissue completely filling the gap between the transected cord stumps 8 weeks after implantation (Fig. 5C).

At 8 weeks after implantation, NSC survival was observed in the conduit+NT-3+NSCs and conduit+NSCs groups. Fluorescent microscopy of frozen sections showed GFP-positive transplanted NSCs in the bridge tissue (Fig. 5D, E). The overall survival of the transplanted NSCs was quantified for each group. The conduit+NT-3+NSCs group exhibited an average GFP-positive cell density of 75.8±15.1%, whereas the conduit+NSCs group exhibited only 32.3±10.2% GFP positive cell density (Fig. 5F). The conduit+NT-3+NSCs group showed significantly improved long-term survival of NSCs compared with the conduit+NSCs group.

**Figure 6:**
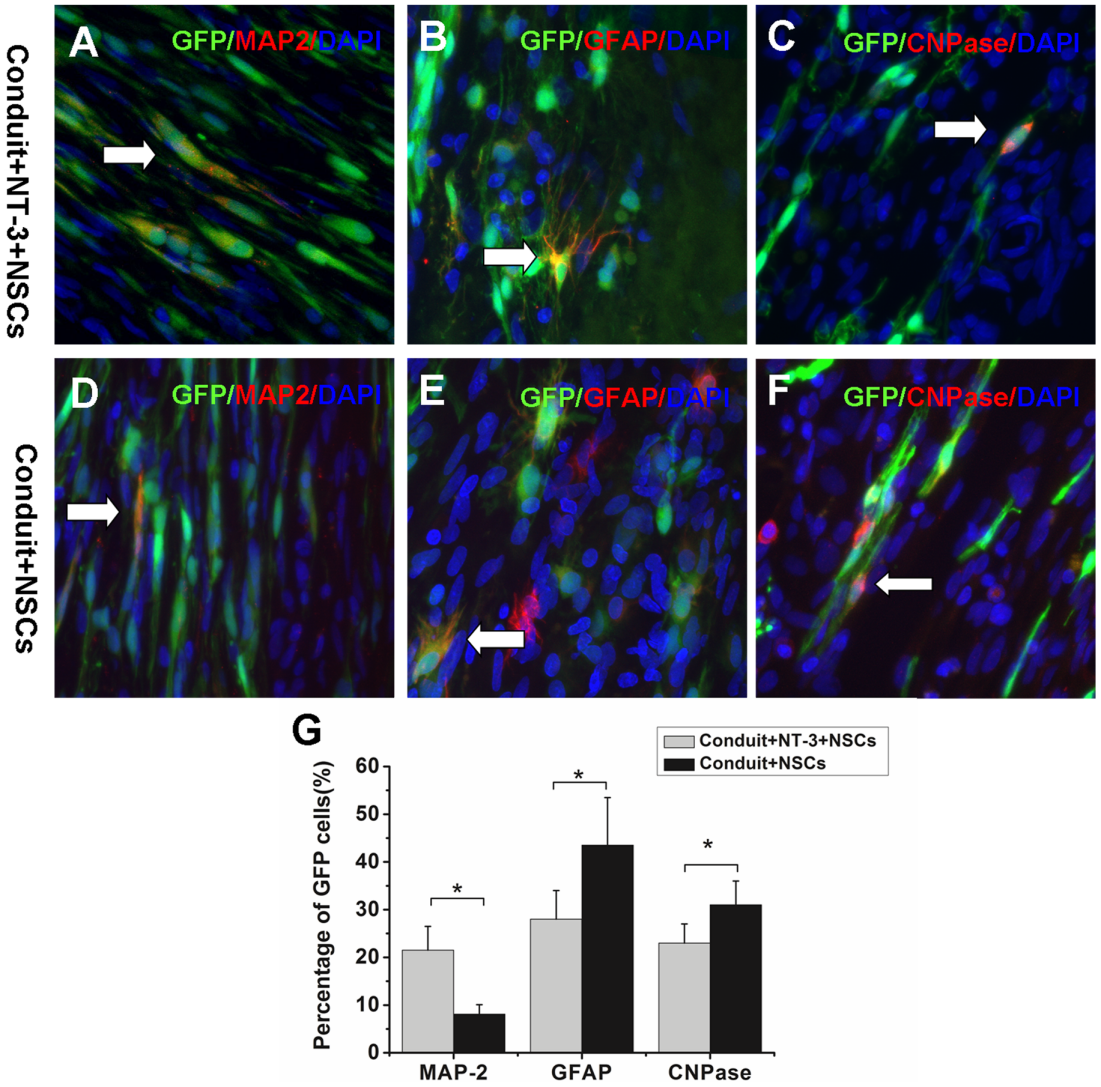
Differentiation profiles of NSCs in vivo. (A–F) Tissue samples were immunostained with MAP-2 for neurons (A, D), GFAP for astrocytes (B, E), and CNPase for oligodendrocytes (C, F) from the conduit+NT-3+NSCs (A–C) and conduit+NSCs groups (D–F). White arrows indicate the representative differentiated cells. (G) Quantification of NSC differentiation profile for each group. Scale bar  =  20 µm. *P<0.05; n = 7.

NSC differentiation after 8 weeks was examined. Immunofluorescent images showed that GFP-positive cells co-localized with MAP-2 positive mature neurons, GFAP-positive astrocytes, or CNPase-positive oligodendrocytes of the conduit+NT-3+NSCs and conduit+NSCs groups (Fig. 6A–F). The results are summarized in Fig. 6G. MAP-2 staining for neurons showed that 21.5±5.2% neuronal differentiation of NSCs in the conduit+NT-3+NSCs group, which was higher than that in the conduit+NSCs group. In contrast, the percentages of GFAP-positive cells and CNPase-positive cells in the conduit+NT-3+NSCs group were lower, compared with the conduit+NSCs group.

### Axon regeneration

**Figure 7:**
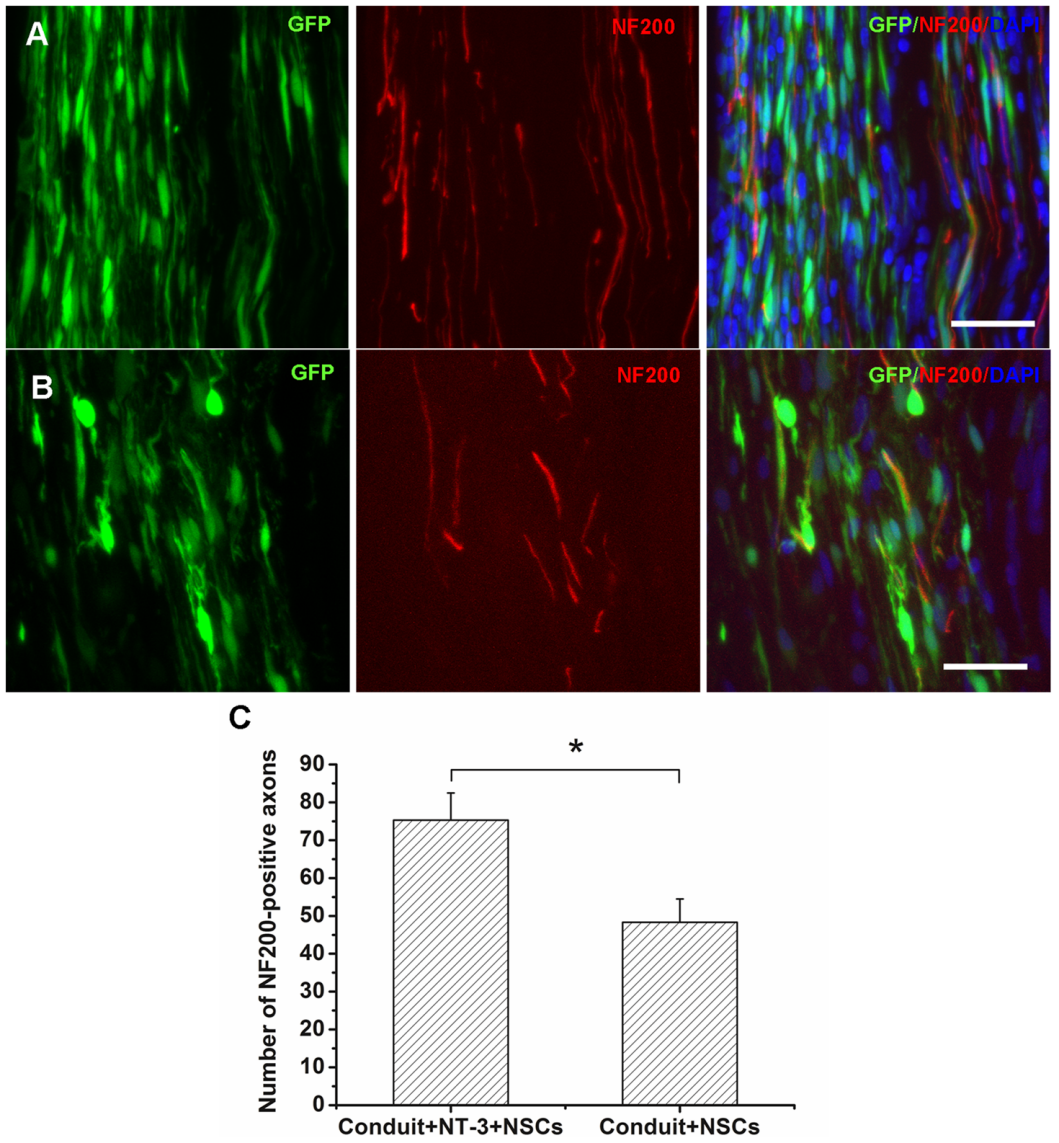
Axonal regeneration. NF200-positive axons were observed in the conduit+NT-3+NSCs (A) and conduit+NSCs (B) groups 8 weeks after implantation. (C) Histograms showing the number of NF200-positive axons for each group. Scale bar  =  20 µm, *P<0.05; n = 7.

Axon regeneration across the bridge was identified by NF200 staining at 8 weeks after implantation. Fluorescent microscopy showed that the regenerating axons were visible in the conduit+NT-3+NSCs group, whereas few regenerating axons were found in the conduit+NSCs group (Fig. 7A and B). The number of NF-200 positive axons in the NSCs contained conduit was determined to be 75.3±7.2 in the conduit+NT-3+NSCs group, and 48.3±6.2 in the conduit+NSCs group. The difference in the number of NF200-positive axons between the two groups was statistically significant (Fig. 7C).

### Locomotor function recovery

Animals were monitored weekly for hindlimb function using the BBB open field locomotor scale. BBB scores were 0 when the spinal cord was completely transected. A time-dependent increase in the BBB scores was displayed in the conduit+NT-3+NSCs, conduit+ NSCs, and conduit+NT-3 groups.

**Figure 8:**
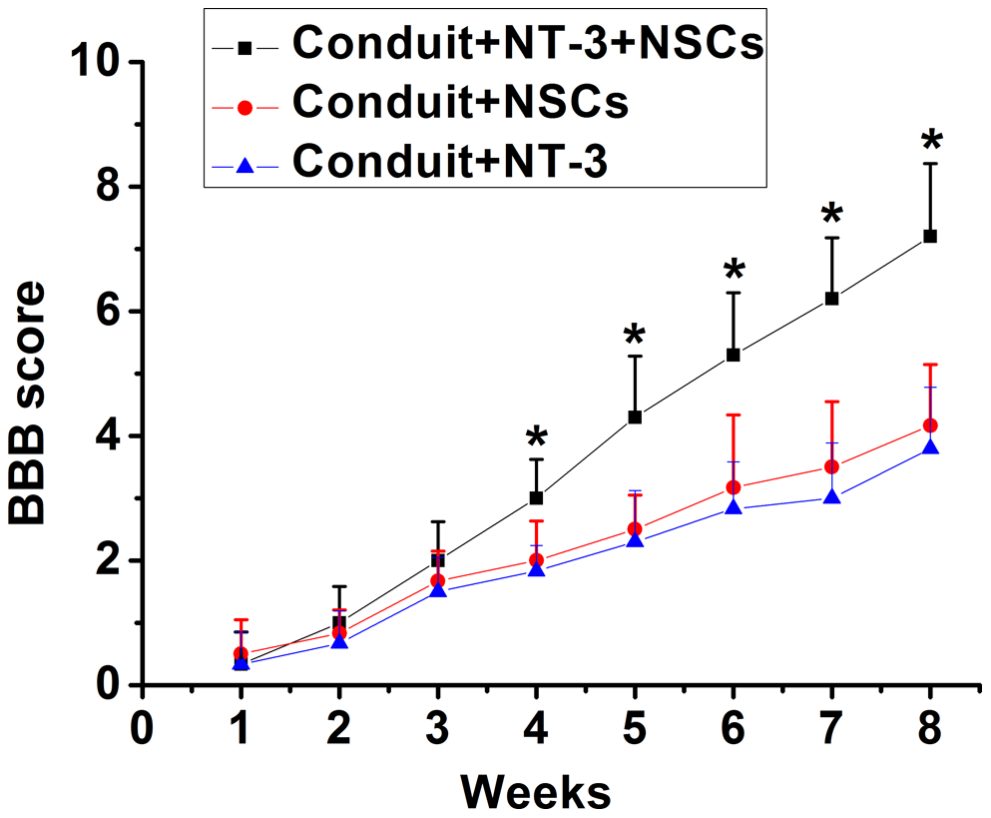
BBB locomotor scores were evaluated weekly for 8 weeks after SCI. At 4 weeks and thereafter, the conduit+NT-3+NSCs group displayed significant behavioral improvement compared with the other two groups. *P<0.05; n = 6.

No significant difference was observed among the three groups until 3 weeks. However, at 4 weeks and thereafter, the conduit+NT-3+NSCs group displayed significant behavioral improvement compared with the other two groups (Fig. 8).

## Discussion

NSCs-based transplantation therapy is currently considered a potentially useful approach for SCI treatment [26]. A major challenge in NSCs-based transplantation therapy is to increase the rate of survival and neuronal differentiation of grafted NSCs. NT-3 is one of the best candidates in stimulating the survival and differentiation of NSCs. However, NT-3 has a short half-life and easily diffuses through tissue and cerebrospinal fluid. Moreover, maintaining a sufficient concentration of NT-3 at the injury site to elicit an effect is difficult [27]. In the present study, we utilized SF β-sheet formation to immobilize NT-3 within the PCLA scaffolds, in which the bioactivity of NT-3 can be maintained, and its release can be controlled for 8 weeks. We then investigated the effects of NT-3-immobilized scaffolds on the survival and neuronal differentiation of NSCs in vitro and in a rat spinal cord transection model. The results showed that the NT-3-immobilized scaffolds enhanced the survival and neuronal differentiation of NSCs in vitro and 8 weeks after implantation in rats. Functional recovery and regeneration of NF200-positive axons were also promoted.

Maintaining the integrity and activity of NT-3 is critical for effective NT-3 delivery. In this study, NT-3 ELISA kits were used to quantify the release of growth factors from the conduits. The release profiles showed that the release of bioactive NT-3 was sustained over 8 weeks. The sustained release is due to the proteolytic degradation of SF coating [28]. The rate of NT-3 release was highest in the first 7 d, and NT-3 release continued at a slower rate for up to 8 wks. This can be ascribed to the inactive of released NT-3 in PBS during the testing interval, and ELISA can only detect the intact NT-3. In the present study, a daily testing interval was initially set, and then the testing interval was changed to weekly.

Currently, the development of biomaterials for neural tissue regeneration and stem cell implantation is a prominent research focus in regenerative medicine. SF has been shown to have excellent biocompatibility both in vitro and in vivo, good mechanical properties, and a slow biodegradation rate [20]. The crystal structure of SF is composed of hydrophilic domains (silk I) and hydrophobic domains (silk II). Hydrophobic blocks make up the crystalline regions of SF due to their ability to form intermolecular β-sheets. Methanol treatement dehydrates and destabilizes the unstable silk I state of SF, leading to a switch to the silk II (predominant) conformation, which is more stable and characterized by an increase in β-sheet content [29]. Therefore, by treating the adsorbed SF/NT-3 molecules on the surface of the scaffolds with methanol, the NT-3 molecules are entrapped in the SF coating. In the present study, SF coating was used as NT-3 reservoir, which played an important role in stabilizing the bioactivity of NT-3. No obvious cytotoxicity of the biomaterials could be observed for the NSCs, due to the biocompatibility of the scaffold and the sustained release of NT-3. In our previous work, we also immobilized NGF within nanofibrous nerve conduits in a concentration gradient pattern by SF coating to guide the dorsal root ganglia (DRG) neurite outgrowth [23]. In sum, the NT-3-immobilized PCLA-SF scaffold is a promising NT-3 delivery system, which increases NSC survival.

SCI results in loss of both sensory and motor neuron function, even neuronal damage and death, as well as axonal demyelination. Despite the presence of endogenous NSC populations within the adult spinal cord, the extent of neuronal differentiation from these endogenous precursor cells, even after infusion of exogenous growth factors, is not sufficient to stimulate neuroplasticity, and rescue neuronal loss after SCI [30]. Thus, restoration of the neuron population by cell transplantation therapy is an attractive strategy in constructing a neural network after SCI. In the present study, enhanced survival and differentiation of transplanted NSCs in vitro and in vivo was achieved using the NT-3 delivery system. Each conduit was seeded with NSCs prior to implantation, and cell survival was assayed 8 weeks after implantation. The conduit+NT-3+NSCs group had a higher density of GFP-positive cells, compared with the conduit+NSCs group. Hence, the NT-3-immobilized scaffolds enhanced the survival of the NSCs not only in vitro but also in vivo.

On the NT-3-immobilized membranes, NSCs differentiated into neurons with a significantly high percentage in vitro. However, when the NT-3-immobilized scaffolds were implanted into the injured spinal cord, this differentiation percentage decreased in vivo, yet was still significantly higher than the control group. Despite this lower cell survival in vivo, the NT-3 group still had the highest effect in terms of NF200-positive axon regeneration and functional recovery. Therefore, although this differentiation percentage was lower in vivo than in vitro, the NT-3 delivery system is still meaningful.

Complete spinal cord transection is the most severe injury model and results in loss of hindlimb movement immediately after injury. In the present study, the conduit+NT-3+NSCs group showed a statistically significant improvement in hindlimb function, resulting in some movement of hindlimb joints. Immunohistochemistry showed that the NF200-positive axons were densest across the tissue bridge. Axon regeneration has been demonstrated in several SCI models [31], [32]. We believe that this finding is important because these axons may provide a structural basis for re-establishment of a neuronal network for physiological signal transduction across the injury site. Lu et al. seeded NSCs derived from human fetal spinal cord in growth-factor-containing fibrin to derive neurons [7]. The graft-derived neurons were found to extend large numbers of axons into the host spinal cord in both rostral and caudal directions. These axons in the host spinal cord formed dense bouton-like terminals around the host dendrites and cell bodies. Synapse formation was identified by immunohistochemistry and electron microscopy, which supported the observed enhanced functional recovery [33]. In our study, the enhanced functional recovery in the conduit+NT-3+NSCs group can be ascribed to the relatively high amount of NF200-positive axons.

Remarkable progress in guiding neuronal differentiation has been achieved by priming NSCs to neuron-restricted progenitor cells in vitro and addition of growth factors, including NTs, sonic hedgehog protein, and retinoid acid [34], [35]. NTs, particularly NT-3, are considered to be the most promising strategy for future clinical application. NT-3 could not only facilitate the survival and proliferation of NSCs, but also induce the differentiation of NSCs into neurons. NT-3 acts via its corresponding receptor tyrosine kinases (TrkA, TrkB, and TrkC). NT-3 preferentially binds to TrkC, and then activates the Akt/MAP kinase signal pathway, which is involved in neurotransmitter regulation and neural peptide synthesis [36]. In several investigations, genetically modified NSCs were utilized to overexpress NT-3 to affect the survival and differentiation of surrounding cells. However, gene transduction is limited by various problems, including concerns about the safety and efficiency of gene transfection and the potential adverse effects of exogenous gene expression [37]. In contrast, PCLA-SF has several advantages for NT-3 delivery. First, SF adsorption and β-sheet formation is a simple method to immobilize growth factors. Multiple growth factors can also be immobilized simultaneously with SF coating, which synergistically improve functional recovery. Furthermore, drugs that eliminate the inhibitory environment or enhance the regenerative ability of axons could also be included. Second, the present NT-3 delivery system is safe. Both PCLA and SF are recommended scaffold materials because of their unique features, such as biocompatibility and biodegradability, for tissue engineering for the repair and regeneration of injured tissues [38], [39].

## Conclusions

By incorporating NT-3 into SF coating, we successfully developed an NT-3-immobilized PCLA scaffold as a delivery system. Seeding NSCs within these NT-3-immobilized PCLA scaffolds not only enhanced the survival of grafted cells but also promoted neuronal differentiation both in vitro and in a rat SCI model. At 8 weeks after implantation, the conduit+NT-3+NSCs group showed significant improvement in axon regeneration and functional recovery. Our results suggest that PCLA conduits immobilized with NT-3 have the potential for SCI repair by promoting survival and neuronal differentiation of transplanted NSCs. In addition, SF adsorption and coating are simple methods for immobilizing bioactive molecules. In the future, drugs that eliminate the inhibitory environment and enhance regeneration can be immobilized within the scaffolds for a synergistic effect.
